# Genome Sequence of *Schizochytrium* sp. CCTCC M209059, an Effective Producer of Docosahexaenoic Acid-Rich Lipids

**DOI:** 10.1128/genomeA.00819-15

**Published:** 2015-08-06

**Authors:** Xiao-Jun Ji, Kai-Qiang Mo, Lu-Jing Ren, Gan-Lu Li, Jian-Zhong Huang, He Huang

**Affiliations:** a State Key Laboratory of Materials-Oriented Chemical Engineering, College of Biotechnology and Pharmaceutical Engineering, Nanjing Tech University, Nanjing, Jiangsu, People’s Republic of China; b Engineering Research Center of Industrial Microbiology, Ministry of Education, College of Life Sciences, Fujian Normal University, Fuzhou, Fujian, People’s Republic of China

## Abstract

*Schizochytrium* is an effective species for producing omega-3 docosahexaenoic acid (DHA). Here, we report a genome sequence of *Schizochytrium* sp. CCTCC M209059, which has a genome size of 39.09 Mb. It will provide the genomic basis for further insights into the metabolic and regulatory mechanisms underlying the DHA formation.

## GENOME ANNOUNCEMENT

Docosahexaenoic acid (DHA), one of the most typical omega-3 polyunsaturated fatty acids, has received worldwide attention due to its beneficial physiological functions for humans (1, 2). Its traditional sources are coldwater fish oils. However, the product quality derived from fish oil is dependent on season and position and is affected by ocean pollution. Alternatively, DHA can be obtained from marine fungal oils (lipids).

*Schizochytrium* sp., which was first derived from coastal seawater as a new thraustochytrid by Nakahara et al. (3), has been widely used to produce DHA. It can accumulate up to 50% of its dry weight as lipids, DHA generally constituting 40% or more of these (4). However, the physiological role of DHA to *Schizochytrium* sp. cells has not been elucidated clearly. DHA, in the form of membrane phospholipids, is generally known to play an important role in membrane functions (5). However, only up to 5% of lipids in *Schizochytrium* sp. occur as phospholipids, whereas 90% or more are generally present as triacylglycerols in storage lipids (6–8). Why do *Schizochytrium* sp. cells accumulate more DHA as triacylglycerols instead of as phospholipids? The reason for this is not clear. Furthermore, in *Schizochytrium* spp., the metabolic pathway for DHA biosynthesis (via polyketide synthases) is different from the pathway in other DHA-producing strains (via fatty acid synthases), such as *Thraustochytrium* spp., *Crypthecodinium cohnii*, and so on (9–11).

We therefore sequenced and analyzed the genome of *Schizochytrium* sp. CCTCC M209059, which is an efficient DHA-producing strain stored in the China Center for Type Culture Collection (12, 13), to reveal the metabolic and regulatory mechanisms underlying the DHA formation, both in relation to its physiological functions and its distinctive metabolic pathways.

The genomic DNA of the strain was isolated using the EZNA fungal DNA kit (Omega Bio-tek, Doraville, GA, USA). The purity and concentration of the DNA were measured by a Qubit Fluorometer (Invitrogen, Carlsbad, CA, USA). Genome sequencing was conducted using the Illumina HiSeq2000 DNA sequencing platform at BGI-Shenzhen (Shenzhen, China). The raw sequence data comprise a total of 61,950,544 reads, assembled into 1,608 contigs through the SOAPdenovo alignment tool (14, 15). The genome assembly is 38,297,968 bp, with an *N*_50_ equal to 52,007 bp, an *N*_90_ of 14,718 bp, and a maximum contig size of 236,430 bp. The contigs were further scaffolded with a total of 39,089,698 bp, which were placed in the final 322 scaffolds, with an *N*_50_ of 595,797 bp, an *N*_90_ of 144,465 bp, and a maximum scaffold size of 1,674,554 bp. This resulted in a predicted genome size of 39.09 Mb with a G+C content of 56.6%. There are a total of 9,142 predicted protein-coding genes, 178 tRNA genes, and 31 rRNA genes in the genome. Annotation of the genome was performed via Gene Ontology Analysis (16) and the Kyoto Encyclopedia of Genes and Genomes pathway database (17). The genome sequence could provide opportunities for investigating the metabolic and regulatory mechanisms underlying the formation of DHA, which will provide guidance for efficient DHA production with high content and high productivity.

### Nucleotide sequence accession numbers.

This whole-genome shotgun project has been deposited at DDBJ/EMBL/GenBank under the accession number JTFK00000000.The version described in this paper is the first version, JTFK01000000.
